# Pork Quality of Two Lithuanian Breeds: Effects of Breed, Gender and Feeding Regimen

**DOI:** 10.3390/ani11041103

**Published:** 2021-04-12

**Authors:** Violeta Razmaitė, Remigijus Juška, Raimondas Leikus, Virginija Jatkauskienė

**Affiliations:** Animal Science Institute, Lithuanian University of Health Sciences, R. Žebenkos 12, LT-82317 Baisogala, Radviliškis District, Lithuania; remigijus.juska@lsmuni.lt (R.J.); raimondas.leikus@lsmuni.lt (R.L.); Virginija.Jatkauskiene@lsmuni.lt (V.J.)

**Keywords:** carcass, meat quality, fatty acids, lipid indices, pig breeds

## Abstract

**Simple Summary:**

A few highly selected commercial pig breeds have been developed and many different local breeds have been replaced by these modern breeds. With the aim to preserve rare local breeds, the necessity has arisen to find and create the conditions under which local breeds could be more widely used. Therefore, the performance and production qualities of local breeds should be evaluated and highlighted. As the quality of pig production is associated with breeds and effects of other different conditions, the objective of this experiment was to investigate the effects of breed, gender, and feeding level in the finishing phase on the carcass and meat quality of Lithuanian White and Lithuanian Indigenous Wattle pigs reared indoors. Feed restriction during the finishing phase and gender both affected the growth and fatness of pigs. The breed and gender appeared to have an effect on the parameters of meat quality including fatty acid composition. The breed and gender effects on fatty acid composition showed more favorable lipid indices in relation to healthy nutrition in the ham muscles of Lithuanian White pigs compared with Lithuanian Indigenous Wattle pigs and also in the ham muscle and backfat of females compared with castrated males.

**Abstract:**

The diversity of breeds is an important factor influencing carcass and meat quality traits that are also associated with other different effects. The objective of this study was to investigate the effects of breed, gender, and feeding level in the finishing phase on the carcass and meat quality of Lithuanian White and Lithuanian Indigenous Wattle pigs reared indoors. After 60 kg weight, half of the animals from both breeds were fed a restricted diet of approximately 82% of average ad libitum feeding intake, and the other half of pigs were further fed ad libitum to the end of the experiment. Feed restriction during the finishing phase decreased daily gain and weight of pigs at slaughter, and backfat thickness at the tenth rib (*p* ˂ 0.001, *p* ˂ 0.01, and *p* ˂ 0.05, respectively). Lithuanian White pigs demonstrated higher (*p* ˂ 0.001) growth rate, live body weight at slaughter and carcass weight, and had a higher (*p* ˂ 0.01) length of carcass and loin area and lower (*p* ˂ 0.05) backfat thickness at the last rib and at two points at the lumbar area compared with Lithuanian Indigenous Wattle pigs. The semimembranosus muscle of Lithuanian White pigs had relatively (8.1%) lower (*p* ˂ 0.001) proportions of saturated and 41.2% higher (*p* ˂ 0.001) proportions of polyunsaturated fatty acids compared with the pigs of the Lithuanian Indigenous Wattle breed, whereas in the longissimus muscle and subcutaneous tissue, the breed only affected the n-6/n-3 ratio (*p* ˂ 0.01 and *p* ˂ 0.001, respectively). Gender showed an effect on saturated fatty acids in all the studied tissues, however, the effects on polyunsaturated fatty acids were found in the semimembranosus muscle and subcutaneous tissue (*p* ˂ 0.05 and *p* ˂ 0.001) and the effects on monounsaturated fatty acids was found only in the semimembranosus muscle (*p* ˂ 0.01). These differences in the fatty acid composition of the semimembranosus muscle and subcutaneous tissue of females exhibited more favorable lipid quality indices compared with castrated males (*p* ˂ 0.001). The semimembranosus muscle of Lithuanian White pigs also showed more favorable lipid quality indices in relation to healthy nutrition compared with Lithuanian Indigenous Wattle pigs (*p* ˂ 0.001). This study is one of the steps toward the development and utilization of endangered breeds. The obtained information can be used to increase choice in pork production and consumption, and provides new insights for research into the conservation of local breeds.

## 1. Introduction

Pig performance, represented by the growth rate, feed conversion efficiency, and lean meat content in carcasses, has been improved in conventional breeds by intensive selection. However, intensive selection has resulted in different negative side effects including poorer meat quality and lower resistance to pig diseases [1,2,3,4]. It is difficult to achieve good meat quality by traditional selection because most meat quality traits exhibit low to moderate heritabilities, so measuring them is difficult, expensive, and mostly possible after slaughter [5,6]. As meat quality has a multifactorial background and can be associated with monogenic and polygenic effects, these effects comprise the differences among breeds [5,6]. Therefore, the breed and diversity of breeds is one of the important factors influencing meat quality traits. Meat quality is one of the most important features for the pork consumer because they expect to be able to purchase and consume meat that meets their requirements for tenderness, juiciness, and flavor [7]. Although meat flavor is a complex attribute, the basis for meat flavor is established in the primary production phase through the choice of breed [8]. Unselected local pigs with poor productive traits, but with good adaptation to environmental conditions, more frequently demonstrate higher meat quality [9,10] and there is a growing interest toward the use of local pig breeds in Europe. Moreover, there is an opinion that the meat of some autochthonous pigs may even be considered as a functional food [11]. The breeds that have almost disappeared are currently being used in the pork chain for fresh meat and meat products in both extensive and intensive farming in South Europe [12,13,14,15,16]. Some different local pig breeds are used not only in the Mediterranean area, but also in other countries [17,18].

Despite the fact that both Lithuanian pig breeds—Lithuanian Indigenous Wattle and Lithuanian White—are close in their origin [19,20] have genetic relationships confirmed at the genomic level [21,22], and are both suitable as dam breeds, they are different breeds with regard to their phenotypic characteristics and selection. Lithuanian White pigs were developed in the 20th century under purposeful selection in the process of improving local pigs using Large White, Middle White, Edelsweine, Berkshire, and local Danish pigs [20], whereas no selection was carried out for old Lithuanian Indigenous Wattle pigs. In addition to characteristic wattles under the neck, Lithuanian Indigenous Wattle pigs usually have black spots on the body and include not only black and white but also other color variations. These pigs are insensitive to the sun [19]. A few decades ago, the Lithuanian White pig breed was one of the main pig breeds used in Lithuania. Nowadays, purebred remains of both above breeds are under a conservation program and need their wider usage.

Evaluation of the breeds and their combinations bred in Lithuania showed lower performance of Lithuanian White pigs compared to conventional breeds [23], however, another study [24] on the longissimus muscle showed their meat quality to be the best among different breeds. Carcass and meat quality studies of Lithuanian Indigenous Wattle pigs demonstrated that cross-breeding with lean breeds [25] or slaughter at lower (60–80 kg) weight [26] could decrease their fatness, but until now, there have been no detailed comparisons of carcass and meat quality traits between the two local Lithuanian breeds. The objective of this study was to investigate the effects of breed, gender, and feeding level in the finishing phase on the carcass and meat quality of Lithuanian local pig breeds reared indoors.

## 2. Materials and Methods

### 2.1. Animals, Diets, and Slaughtering

The experiment included pigs from two local Lithuanian breeds: 18 Lithuanian Indigenous Wattle and 24 Lithuanian White old genotype. The experimental groups comprised piglets with an average initial weight of 30 kg. The pigs were kept in gender-mixed (females and castrated males) single–breed groups of four pigs per pen with a 1.75 m^2^ space allowance per pig on a solid straw bedded floor, and fed the same feed for all groups ad libitum according to their appetite on conventional concentrates containing 16.8% crude protein, 2.18% fat, and 12.4 MJ metabolizable energy (ME) up to 60 kg live weight. The major compounds of feedstuff were barley (31.4%) and triticale (30%), wheat (15%), rapeseed cake (8.5%), soy meal (6.5%), and sunflower meal (5%). After 60 kg weight, half of the animals from both breeds were fed a fixed restricted diet of approximately 82% of the average ad libitum feeding intake, and the other half were further fed ad libitum to the end of the experiment. Due to the differences between the breeds in pig appetite, feed consumption was different (Table 1).

Slaughtering was conducted in the abattoir for control slaughtering at the state pig breeding station after 4 km (10 min trip) with minimum handling stress. Lairage time was approximately 2.5 h. Pigs were electrically stunned (head-only; 1.3 A, 240 V for 3–4 s) and bled within 15 s of stunning. Afterward, the carcasses were scalded in a Hubert H_AAS_ scalder (Neuler, Germany) for 3–4 min at 65–70 °C and split longitudinally on the midline within 20–25 min post mortem.

### 2.2. Carcass Evaluation and Meat Sampling

Eviscerated carcasses with head and legs were weighed to determine the warm carcass weight and chilled overnight at +2–4 °C. The dressing percentage was calculated as the percentage of carcass weight 30 min after slaughtering on live weight before slaughter. After 24 h post-mortem, the carcasses were weighed again to determine cold carcass weight. The cooling loss was calculated as the difference between warm and cold carcass weight in percentage.

The measurements of backfat depth were taken with a ruler on the left side of carcasses at the dorsal midline at the first, tenth, and last ribs, and at three points in the lumbar area: before, above, and behind *M. gluteus.* The measurements of the ventral part of belly thickness were carried out along the line of teats at three points: 5 cm caudally from the last teat, parallel with the last thoracic vertebra, and 3 cm caudally from the sternum and striked an average.

The left side of each carcass was weighed, cut into four anatomically defined primal cuts (ham, shoulder with neck, loin, and belly) according to Metayer and Daumas [27], and all parts were weighed. Photos were taken to measure the area of *M. longissimus dorsi* (LD) at 1/2 lumbar vertebra by an EX-Z110 (Casio, Tokyo, Japan) digital camera. Afterward, the loin area was planimetrically measured by means of the “SCAN-STAR K” planimetrical system (Ingenieurbüro R. Matthäus, Nobitz, Germany). The samples of LD were collected from the loin of the left side of the carcasses. To analyze the technological meat quality, the samples of the posterior part of LD were taken at the 1/2 lumbar vertebra. The samples of *M. semimembranosus* (SM) were collected from the ham of the left side of the carcasses.

### 2.3. Meat Quality Assessments

#### 2.3.1. Chemical Composition of Muscles

The dry matter content was determined [28] by drying samples in an oven at 105 °C until a constant weight was obtained (method No 950.46B; AOAC, 1990). The crude protein content was determined by the Kjeldahl method using the Kjeltec system 1002 apparatus (Foss-Tecator AB, Höganäs, Sweden), and a conversion factor of 6.25 was used to convert total nitrogen to crude protein (method no. 981.10; AOAC, 1990). Crude fat was determined by the Soxhlet extraction method (method no. 960.39; AOAC, 1990). Ash was determined by incineration in a muffle furnace at 550 °C for 24 h (method no. 920.153; AOAC, 1990). The content of protein, fat, and ash were expressed as the weight percentage of dry matter from muscle tissues.

The hydroxyproline content was determined by the NMKL-AOAC method [29].

The cholesterol content in meat was determined according to the extraction method described by Polak et al. [30] and followed by a high-performance liquid chromatography (HPLC) separation and analysis on a Shimadzu 10 A HPLC system (Shimadzu Corp., Kyoto, Japan). The data collection and evaluation were performed by using a LC Solution (Shimadzu Corp., Kyoto, Japan) operating system. The analytical column was LiChrospher 100 RP-18e, 150 × 4.6 mm, 5 μm (Alltech Associates Inc., Chicago, IL, USA) with a guard column (LiChrospher 100 RP-18, 7.5 × 4.6 mm). The cholesterol content was expressed as mg/100 g fresh meat.

#### 2.3.2. pH Measurements

pH was measured using a digital portable pH-meter PT-380 (Boeco, Hamburg, Germany) equipped with a glass electrode (Witeg Laboratory Technik GMBH, Wertheim, Germany) after calibration using pH 4.0 and 7.0 buffer solutions. pH1 was determined 30–45 min post-mortem directly on the carcass before cooling. Ultimate pH (pHu) was determined 24 h after slaughter. In LD muscle, pH was determined between the 13th and 14th last ribs at a 5 cm depth and in SM muscle, pH was determined 5 cm above the bone and at 3 cm depth.

#### 2.3.3. Color

The color parameters were measured using a chromameter CR-410 Konica Minolta (Osaka, Japan) equipped with a 50 mm aperture using a C illuminant and 2° standard observer calibrated to a standard white calibration plate (Y = 85.3, x = 0.3173, y = 0.3251) in the CIE L* a* b* and L* C h color spaces (lightness, L*; redness, a*; yellowness, b*; chroma, C, and hue, h) on the fresh cut surface after 30 min blooming at room temperature (18 °C).

#### 2.3.4. Water Holding Capacity

Water holding capacity was measured in two ways: drip loss and cooking loss. The drip loss was assessed according to the EZ-DripLoss method [31]. To determine cooking loss, the frozen samples for texture profile analysis (TPA) were thawed at 4 °C for 24 h, weighed, and cooked in thin walled plastic bags at 80 °C for 1 h by immersion in a water bath with automatic temperature control [32], and then cooled at room temperature (18 ± 2 °C) and weighed again. The cooking loss (%) is defined as the difference in weight of the sample (after wiping dry) before and after cooking and cooling, divided by the sample weight at the beginning and multiplied by 100.

#### 2.3.5. Instrumental Evaluation of Texture

The tenderness of LD and SM muscles was instrumentally measured by the Warner–Bratzler shear test (WB) using a Texture Analyzer TA 1 (Measurement and Calibration Technologies Ametek Comp., Lloyd Instruments, Largo, FL, USA) after cooking and cooling at room temperature (20 °C). The samples for the WB test were obtained by cutting rectangles of 2 × 2 cm in cross section, parallel to the muscle fiber direction. These were completely cut using a WB shear blade with a triangular slot cutting edge and two parameters were measured: work of shear and toughness, according to the following testing procedures: pre-test speed, 3 mm/s; test speed, 1 mm/min; and post-test speed, 3 mm/s. The tenderness was measured and calculated in Newtons (N) using Lloyd Instruments Ltd. Nexygen/Ondio software together with Production Test program Version V3.0.1.

### 2.4. Fatty Acid Profiles

The extraction of lipids for fatty acid analysis was performed with a mixture of two volumes of chloroform (Chromasolv Plus for HPLC containing 0.5–1.0% ethanol as a stabilizer) and one volume of methanol as described by Folch et al. [33]. Methylation of the samples was performed using sodium methoxide: 5 mL of 25 wt % solution in methanol was added to the sample and stirred. After 1 h, 7 mL HCL, 6 mL hexane, and 2 mL H_2_O were added. The top layer was transferred into a new test-tube and evaporated. Fatty acid methyl esters were prepared according to the procedure described by Chritopherson and Glass [34]. The FAMEs were analyzed using a gas liquid chromatograph (GC-2010 SHIMADZU, Kyoto, Japan) fitted with a flame ionization detector. The separation of the methyl esters of fatty acids was affected on the capillary column Rt 2560 (100 m × 0.25 mm × 0.2 μm; Restek, Bellefonte, PA, USA) by temperature programming from 160 °C to 230 °C. The temperatures of the injector and detector were held at 240 °C and 260 °C, respectively. The rate of flow of carrier gas (nitrogen) through the column was 0.79 mL/min. The peaks were identified by comparison with the retention times of the standard fatty acids methyl esters “37 Component FAME Mix” and trans FAME MIX k 110 (Supelco, Bellefonte, PA, USA). The relative proportion of each fatty acid was expressed as the relative percentage of the sum of the total fatty acids using “GC solution” software for Shimadzu gas chromatograph workstations.

#### Lipid Quality Indices

Lipid quality indices (i.e., atherogenic index (AI) and thrombogenic index (TI), were calculated according to Ulbricht and Southgate [35]. The hypocholesterolemic/hypercholesterolemic (h/H) ratio was calculated according to Santos-Silva et al. [36]. The peroxidizability index (PI) was determined according to Du et al. [37].

### 2.5. Statistical Analysis

The data were subjected to the analysis of variance in the general linear (GLM) procedure in IBM SPSS Statistics 22 with LSD tests to determine the significance of differences of means between the groups. The GLM model included fixed factors of breed, gender, feeding level, and factor interactions (breed × gender; breed × feeding level and gender × feeding level). The weight of pigs was included as a covariate. The differences were regarded as significant when *p* ˂ 0.05, but the differences of 0.05 ≤ *p* ˂ 0.10 would be considered as trends.

## 3. Results and Discussion

### 3.1. Growth Rate of Pigs and Carcass Traits

The growth rate of pigs (Table 2) was affected by the breed, feeding level, and gender (*p* ˂ 0.001, *p* ˂ 0.001, and *p* ˂ 0.05, respectively). Lithuanian White pigs reached slaughter about nine days younger (*p* ˂ 0.05) with 9.9 kg heavier (*p* ˂ 0.001) live body weight compared to Lithuanian Indigenous Wattle pigs. The differences between the growth performance of different pig breeds were reported by other authors [38,39,40,41].

Higher pig weight demonstrated increased carcass weight, length, and higher dorsal fat thickness at the tenth rib (*p* ˂ 0.001, *p* ˂ 0.01, and *p* ˂ 0.05, respectively).

Lithuanian White pigs tended to show heavier (2.8 kg; *p* = 0.071) carcasses than Lithuanian Indigenous Wattle pigs as a consequence of their higher live body weight (Table 3).

Lithuanian White pigs were not lean as they were selected both for bacon and for heavy and fatty conditions of slaughter pigs [42]. However, the breed affected the length and fatness of the carcass: Lithuanian White pigs had 6.8 cm longer (*p* ˂ 0.01) carcasses with lower (*p* ˂ 0.05) backfat depth at the last rib and at two points of the lumbar area and larger (*p* ˂ 0.01) loin area compared with Lithuanian Indigenous Wattle pigs.

Similar results were reported by Polish authors [43] who compared the carcass quality of native Pulawska pigs with Polish Large White and found that the carcass weight and length, and the loin area of Pulawska pigs were lower, and backfat thickness was higher compared with Polish Large White.

The feeding level during the finishing phase in the present study also affected the live body and carcass weights and depth of backfat at the tenth rib (*p* ˂ 0.001).

Similar feed restriction effects on carcass weight and fatness have been reported by many authors [43,44,45,46,47]. However, other authors [48] have reported lower pig growth during feed restriction without any effect on backfat depth. Females and castrated males exhibited similar live body, carcass weight, and length as well as dressing, but females slightly tended to show lower (*p* = 0.096) depth of backfat at the tenth rib compared with castrated males. Lower fatness of female carcasses is in agreement with the findings of Serrano et al. [49,50].

According to literature sources [51], substantial changes in the proportional weights of carcass cuts are obtained for loin and belly during the growth of pigs.

In the present study, the heavier carcasses were found for pigs fed without restriction at the finishing stage (Table 4). However, only Lithuanian White tended to show anincrease inthe shoulder percentage compared with Lithuanian Indigenous pigs (*p* = 0.057). Polish local Pulawska pigs showed lower weight and percentage of ham in the carcass than Large White [43]. The differences between Lithuanian pig breeds in the proportions of carcass primal cuts were lower than those between autochthonous Italian Casertana and Large White, and commercial Duroc pigs [39]. Low feeding regimen for Mexican hairless pigs together with their age increase at slaughter increased the yield of all primal cuts [52].

Feeding restriction at the finishing phase also tended to increase (*p* = 0.061) the proportion of belly. There were no differences in the proportions of carcass primal cuts between males and females.

### 3.2. Meat Quality

Meta-analysis [53] identified significant breed effects on pork quality traits, with large variation in the actual magnitudes of breed effects among individual traits. In the present study, the breed showed an effect on the dry matter content in both muscles (*p* ˂ 0.05; Table 5). Lithuanian White pigs also had higher (*p* ˂ 0.01 and *p* ˂ 0.05, respectively) content of protein and ash in LD muscle, and more fat in SM muscle (*p* ˂ 0.05). Pig selection for high lean growth reduces intramuscular fat content [54], and the higher the leanness of pigs, the lower an intramuscular fat content is found [55]. This finding can be considered to be consistent with the results of Florowski et al. [56], who reported that local breeds had higher content of intramuscular fat compared with Landrace. Although Lithuanian Indigenous Wattle pigs are characterized by thick fat cover and a large amount of intermuscular fat, however, their intramuscular fat (IMF) is lower compared with less fatty Lithuanian White pigs. As the weight of pigs increased from 90 to 120 kg, IMF also increased from 1.51 to 2.92% in SM of Lithuanian White pigs (*p* ˂ 0.05). However, when the weight increased from 90 to 110 kg, the increase in IMF in both muscles of both breeds was insignificant. The heaviest (above 110 kg) Lithuanian White pigs had insignificantly lower (2.12%) fat content in LD compared with pigs (2.24%) weighing 100.1–110 kg.

Lower (*p* ˂ 0.001) cholesterol contents were found in the backfat than in the muscles of both Lithuanian breeds, and this is in agreement with the authors [57] who reported that backfat from adult pigs had lower cholesterol content in comparison with muscles.

The breed and gender appeared to show their effects on the cholesterol contents in the backfat and SM muscle, respectively (*p* ˂ 0.05; Table 5). Lithuanian White pigs showed higher cholesterol content in the backfat compared to Lithuanian Indigenous Wattle pigs. The cholesterol content in SM of females was higher than that of castrated males.

Muscle and pH_45 min_ was higher (*p* ˂ 0.001) in SM of Lithuanian Indigenous Wattle pigs than in Lithuanian White pigs (Table 6). Chromameter redness (a*) of LD and SM muscles was higher (*p* ˂ 0.05 and *p* ˂ 0.01, respectively) in Lithuanian White than in Lithuanian Indigenous Wattle pigs. Lithuanian White pigs also showed higher (*p* ˂ 0.05) chroma (C) and tended to show lower (*p* = 0.084) lightness (L*) and higher (*p* = 0.078) thawing loss in SM compared with Lithuanian Indigenous Wattle pigs.

Despite the declared breed effects on pork quality traits [16,38,53,58,59], the differences between the analyzed meat quality parameters for both Lithuanian breeds were greater when comparing their different muscles than the same muscles between the breeds. LD showed higher (*p* ˂ 0.001) color lightness, lower (*p* ˂ 0.001) redness and chroma, higher (*p* ˂ 0.001) hue angle, and lower (*p* ˂ 0.001) cooking loss in both breeds compared with SM, whereas Gil et al. [59] reported that the characteristics of these muscles from different origin pigs were related only to the differences in drip loss. SM of Lithuanian Indigenous pigs also showed higher (*p* ˂ 0.01) toughness. The obtained differences between the muscle parameters were also in contrast with the findings of Lebret et al. [60], who reported slight differences between the muscles.

Castrated males tended to show higher (*p* = 0.062) LD muscle pH_45 min_ than gilts, and this is in agreement with the report of Egea et al. for the Iberian × Duroc cross [61]. Alonso et al. [62] found that castrated males of conventional breeds had more intense meat color than gilts.

However, neither the feeding intensity at the finishing stage nor the gender of Lithuanian pigs had any effect on other meat quality characteristics. This is in agreement with the findings of Serrano et al. [49], who reported no influence of Iberian pig gender on meat quality, but were in contrast with the findings on Korean Native Black pig, where the castrates showed lower values of color measures and cooking loss compared with gilts [63].

### 3.3. Fatty Acid Composition

Although the species is the major source of variation in meat fatty acid composition [64], genetic variability also consists of the differences between breeds and lines, and of the differences between animals within breeds. The pigs may strongly differ concerning their carcass and meat fat contents as well as the anatomical location of tissues. Moreover, pigs are amenable to changes in the fatty acid composition using different diets and feeding regimen [64,65,66]. In the present study, the feeding regimen at the finishing stage did not affect the fatty acid composition either in the muscles or subcutaneous tissue and this is in contrast with the findings of other authors [67,68,69] who have reported fatty acid composition change at restricted feeding. However, some authors have reported that a restricted diet did not demonstrate great differences in the fatty acid composition between different skeletal muscles [67,70]. A high initial and low final feeding level applied for Iberian pigs [71] also did not show any differences in the subcutaneous fatty acid composition.

The effects of breed and gender on the fatty acid composition in LD muscle were minimal (Table 7). Although breed effects on the fatty acid composition in LD were reported for different breeds [72,73], small differences between Lithuanian breeds were found only for some individual fatty acids, consequently resulting in lower (*p* ˂ 0.01) n-6/n-3 PUFA ratio in LD of Lithuanian White pigs compared with Lithuanian Indigenous Wattle. The gender of pigs appeared to show effects on the percentage of total saturated fatty acids (SFA) and some minor individual fatty acids. Castrated males had relatively 3.7% higher (*p* ˂ 0.05) proportion of SFA in LD than gilts. There was a significant interaction for SFA between the breed and gender (*p* ˂ 0.01). Lithuanian White gilts had lower percentage of saturated fatty acids than Lithuanian Indigenous Wattle castrates.

The effects of the breed and gender in SM were significantly higher than those found in the LD. As fatty acid composition is associated with both the overall pig fatness and the fat content in muscles [64,65], larger differences in the fatty acid composition of SM can be explained by larger IMF differences both between the breeds and between LD and SM muscles. SM of Lithuanian White pigs had relatively 8.1% lower (*p* ˂ 0.001) proportions of total saturated fatty acids (SFA) and 41.2% higher (*p* ˂ 0.05) proportions of total polyunsaturated fatty acids (PUFA) including n-3 and n-6 PUFA compared with the pigs of the old Lithuanian Indigenous Wattle breed (*p* ˂ 0.01 and *p* ˂ 0.05, respectively). Other authors [62,67,74] have also indicated significant effects of breed on the fatty acid composition in SM muscle.

Similar effects on the fatty acid composition in SM lipids were also shown by pig gender. Castrated males had higher (*p* ˂ 0.001 and *p* ˂ 0.01, respectively) proportions of total SFA and monounsaturated fatty acids (MUFA), but 35.6% lower (*p* ˂ 0.001) proportion of PUFA including n-3 and n-6 PUFA, and a lower (*p* ˂ 0.01) n-6/n-3 PUFA ratio compared with females. The interaction for SFA in SM between the breed and gender showed that Lithuanian Indigenous Wattle gilts had a lower percentage of saturated fatty acids than Lithuanian White castrates (*p* ˂ 0.001). Supplementary data regarding fatty acid composition of intramuscular fat in both different muscles and in subcutaneous fat can be found in Tables S1–S3, respectively.

There were no significant differences in either the individual most abundant fatty acids or the total SFA, MUFA, and PUFA of the subcutaneous tissue between the breeds (Table 7). Compared with Lithuanian Indigenous Wattle, the subcutaneous tissue of Lithuanian White pigs also had higher (*p* ˂ 0.001) proportions of n-3 PUFA and lower (*p* ˂ 0.01) n-6/n-3 PUFA ratio.

The gender of pigs also appeared to show the effect on fatty acid composition in the subcutaneous tissue. Compared with females, castrated males also had higher and lower proportions, respectively, of total SFA and PUFA, including both n-3 and n-6 PUFA (*p* ˂ 0.05). These differences between the genders resulted in a lower (*p* ˂ 0.05) PUFA/SFA ratio in the subcutaneous tissue of castrates compared with gilts, and this is in agreement with the findings of Correa et al. [66] in the fat of pig bellies. There were significant interactions between the breed and gender for MUFA and the n-6/n-3 PUFA ratio in the subcutaneous tissue (*p* ˂ 0.05). Lithuanian White castrated males had a lower proportion of MUFA than Lithuanian Indigenous Wattle females, but Lithuanian White gilts had a higher proportion of MUFA than Lithuanian Indigenous Wattle castrates. Lithuanian White castrated males also showed lower n-6/n-3 ratio than Lithuanian Indigenous Wattle females. The PUFA/SFA ratio (Table 7) in LD muscle and backfat was below the minimum (0.4) recommended for the diet [65]. These ratios in SM were affected by breed and gender and satisfied the recommendations for the consumer diet.

Higher and more favorable PUFA/SFA ratios were found in SM muscle of Lithuanian White pigs and females. These ratios in Lithuanian pork were similar to those for the meat from Polish Pulawska [73], but significantly higher than in the meat of Serbian Mangalitsa and Moravka pigs kept on a different free range system [72]. Recommendations of Bellagio’s report on healthy agriculture, healthy nutrition, and healthy people indicated that the ratio (4:1) of n-6 PUFA to *n*-3 PUFA in the diet should be the goal [75]. It can be observed that n-6/n-3 PUFA ratio in LD of Lithuanian White pigs was lower and more favorable compared with Lithuanian Indigenous Wattle pigs. n-6/n-3 PUFA ratios in the studied muscles and subcutaneous tissue were higher than recommended, however, they were lower and more favorable than those in the pork from different breeds reported by other authors [73,76].

The differences in the fatty acid composition appeared to affect lipid quality indices in the IMF and subcutaneous tissue (Table 8). Lithuanian White pigs showed more favorable lower (*p* ˂ 0.001) atherogenic (AI) and thrombogenic (TI) indexes as well as higher (*p* ˂ 0.001) hypocholesterolemic/hypercholesterolemic (h/H) ratio, but less favorable peroxidizability index (PI) in SM lipids than Lithuanian Indigenous Wattle pigs (*p* ˂ 0.05). Similar AI and TI indexes were reported for Pulawska pigs [73].

Pig gender had the largest effect on lipid quality indices. Castrated males had higher (*p* ˂ 0.05) TI index and tended to show a lower (*p* = 0.059) h/H ratio in the lipids of LD. Less favorable lipid quality indices for commercial crossbred pigs were found by Hanczakowska et al. [76], but the trend of the gender effect was similar. In the present study, castrates were also characterized by higher (*p* ˂ 0.001) AI and TI indexes, and a lower (*p* ˂ 0.001) hypocholesterolemic/hypercholesterolemic (h/H) ratio and peroxidizability index (PI) in the SM lipids than females. Significant interactions were found between the breed and gender for the AI and TI indexes and for the h/H ratio in LD and SM (*p* ˂ 0.01). These indexes and the ratio in LD of Lithuanian Indigenous Wattle gilts were, respectively, lower and higher compared with Lithuanian White castrates. AI, TI indexes and h/H ratio in SM of Lithuanian White females were, respectively, lower and higher compared with Lithuanian Indigenous Wattle castrated males. The subcutaneous tissue of castrated males showed higher (*p* ˂ 0.05 and *p* ˂ 0.01, respectively) AI and TI indexes and a lower (*p* ˂ 0.01) h/H ratio and PI index compared with gilts.

## 4. Conclusions

Lithuanian White pigs demonstrated higher (*p* ˂ 0.001) growth rate, live body weight at slaughter and carcass weight as well as higher (*p* ˂ 0.01) length of carcass and loin area and lower (*p* ˂ 0.05) backfat thickness at the last rib and at two points at the lumbar area compared with Lithuanian Indigenous Wattle pigs.

Feed restriction during the finishing phase decreased daily gain and weight of pigs at slaughter, and backfat thickness at the tenth rib (*p* ˂ 0.001, *p* ˂ 0.01, and *p* ˂ 0.05, respectively).

The breed exhibited the effects on meat color, composition and fatty acid composition. The differences in the fatty acid composition, particularly those of SM muscle of Lithuanian White pigs and SM muscle and subcutaneous tissue of females, exhibited more favorable lipid quality indices compared with Lithuanian Indigenous Wattle pigs and males (*p* ˂ 0.001).

This study is one of the steps toward the development and utilization of endangered Lithuanian breeds. The obtained information can be used to increase the choice in pork production and consumption, and provide new insights for research into the conservation of local breeds.

## Figures and Tables

**Table 1:** Average feed consumption and conversion per pig by breed.

Feed Consumption and Conversion	Feeding Level			
	Ad libitum throughout All Period		Restricted after 60 kg Weight	
	LW	LIW	LW	LIW
Feed consumption per pig, kg				
Before feed restriction	92.7	83.7	92.1	83.1
After feed restriction	183.2	184.6	152.8	148.2
Total feed consumption per pig	275.9	268.3	244.9	231.3
Feed conversion, feed kg/per gain kg				
Before feed restriction	2.70	2.97	2.71	3.19
After feed restriction	4.00	4.50	3.88	4.29
Throughout experiment	3.44	3.87	3.34	3.82

LW—Lithuanian White; LIW—Lithuanian Indigenous Wattle.

**Table 2:** Growth performance and live weight of pigs at slaughter.

Variables	Breed		Gender		Feeding Regimen		SED	*p*-Value		
	LW *n* = 24	LIW *n* = 18	CM *n* = 14	F *n* = 28	Ad lib *n* = 21	R *n* = 21		B	G	FR
Live weight, kg	108.6	98.7	105.2	102.2	107.3	101.1	2.33	˂0.001	0.191	0.003
Age, days	203.5	212.3	210.1	205.6	207.4	208.5	4.16	0.038	0.284	0.792
Daily gain, g	743.3	619.2	704.1	658.4	718.4	644.2	18.66	˂0.001	0.017	˂0.001

LW—Lithuanian White; LIW—Lithuanian Indigenous Wattle; CM—castrated males; F—females; Ad lib—ad libitum; R—restricted; SED—Standard error of difference; B—breed; G—gender; FR—feeding regimen. *p*-values of GLM LSD tests for breed, gender, and feeding regimen were significantly different at *p* ˂ 0.05.

**Table 3:** Effects of breed, gender, and feeding intensity on pig carcass characteristics.

Variables	Breed		SED	Gender		SED	Feeding Regimen		SED	*p*-Value			
	LW *n* = 24	LIW *n* = 18		CM *n* = 14	F *n* = 28		Ad lib *n* = 21	R *n* = 21		B	G	FR	W
Hot carcass weight, kg	78.43	75.67	1.446	77.73	77.19	0.646	77.98	76.80	0.577	0.071	0.417	0.053	˂0.001
Dressing, %	75.88	73.07	1.390	75.23	74.59	0.621	75.47	74.18	0.555	0.058	0.317	0.032	0.095
Carcass length, cm	99.89	93.09	1.940	97.09	97.49	0.976	97.38	97.30	0.902	0.002	0.685	0.929	0.010
Internal fat, kg	1.79	1.75	0.290	1.90	1.70	0.146	1.91	1.64	0.135	0.907	0.189	0.056	0.079
Backfat at 1 rib, mm	48.15	54.01	4.983	52.39	49.12	2.507	52.28	48.41	2.316	0.250	0.204	0.108	0.211
Back fat at 10 rib, mm	28.81	35.71	3.884	33.51	30.13	1.954	33.18	29.48	1.805	0.088	0.096	0.044	0.028
Backfat at last rib, mm	26.57	34.63	3.881	30.63	28.97	1.952	30.96	28.23	1.804	0.048	0.404	0.142	0.069
Lumbar fat 1, mm	30.07	34.30	5.97	32.64	31.07	3.002	33.30	30.01	2.773	0.485	0.605	0.247	0.070
Lumbar fat 2, mm	21.19	33.31	5.480	26.42	25.32	2.76	27.06	24.41	2.547	0.036	0.694	0.308	0.199
Lumbar fat 3, mm	29.67	44.72	7.014	35.55	35.17	3.529	35.48	35.15	3.260	0.042	0.917	0.919	0.107
Mean depth of underbelly, mm	22.78	22.57	2.912	21.93	23.16	1.465	23.60	21.80	1.354	0.944	0.409	0.195	0.105
Loin area, cm^2^	34.26	24.82	3.33	31.66	30.16	1.674	32.05	29.39	1.547	0.009	0.377	0.097	0.056

LW—Lithuanian White; LIW—Lithuanian Indigenous Wattle; CM—castrated males; F—females; Ad lib—ad libitum; R—restricted; SED—Standard error of difference; B—breed; G—gender; FR—feeding regimen; W—weight. *p* values of GLM LSD tests for breed, gender, and feeding regimen were significantly different at *p* ˂ 0.05.

**Table 4:** Effects of breed, gender, and feeding regimen during the finishing phase on primal cuts of pig carcasses.

Variables	Breed		SED	Gender		SED	Feeding Regimen		SED	*p*-Value			
	LW *n* = 24	LIW *n* = 18		CM *n* = 14	F *n* = 28		Ad lib *n* = 21	R *n* = 21		B	G	FR	W
Ham %	21.90	21.51	0.997	21.53	21.89	0.501	21.39	22.34	0.435	0.703	0.476	0.130	0.413
Shoulder %	34.55	31.63	1.474	33.87	33.18	0.741	33.63	33.48	0.591	0.057	0.365	0.586	0.190
Loin %	24.85	24.98	3.536	24.43	25.18	1.779	24.98	24.49	1.300	0.972	0.677	0.925	0.410
Belly %	15.33	16.85	3.012	15.66	16.05	1.515	14.53	16.84	1.192	0.617	0.801	0.061	0.169

LW—Lithuanian White; LIW—Lithuanian Indigenous Wattle; CM—castrated males; F—females; Ad lib—ad libitum; R—restricted; SED—Standard error of difference; B—breed; G—gender; FR—feeding regimen; W—weight. *p*-values of GLM LSD tests for breed, gender, and feeding regimen of 0.05 ≤ *p* ˂ 0.10 would be considered as trends.

**Table 5:** Effects of breed, gender, and feeding regimen during the finishing phase on the chemical composition of pig muscles.

Variables	Breed		Gender		Group		SED	*p*-Value		
	LW *n* = 24	LIW *n* = 18	CM *n* = 14	F *n* = 28	Ad lib *n* = 21	R *n* = 21		B	G	FR
Longissimus muscle										
Dry matter, %	26.36	25.78 ^e^	25.88	26.27	25.96	26.19	0.284	0.050	0.183	0.427
Protein, %	23.04 ^e^	22.34	22.55	22.83	22.69	22.69	0.243	0.007	0.256	0.998
Fat, %	2.14	2.26 ^c^	2.24	2.16	2.12	2.28	0.273	0.643	0.749	0.572
Ash, %	1.02	0.94	0.96	1.00	0.96	0.99	0.030	0.011	0.116	0.305
Hydroxyproline, mg/100 g	42.00 ^c^	43.18	42.19	42.99	39.71	45.47	3.007	0.701	0.793	0.069
Cholesterol, mg/100 g	37.77 ^e^	38.40 ^e^	37.67	38.51	37.17	39.01	1.876	0.742	0.656	0.333
Semimembranosus muscle										
Dry matter, %	26.08	25.08 ^f^	25.64	25.52	25.69	25.48	0.38	0.012	0.758	0.585
Protein, %	22.34 ^f^	22.21	22.20	22.35	22.17	22.38	0.23	0.570	0.509	0.362
Fat, %	2.63	1.54 ^d^	2.23	1.93	2.30	1.86	0.45	0.022	0.517	0.333
Ash, %	1.05	1.02	1.04	1.03	1.03	1.04	0.04	0.538	0.874	0.758
Hydroxyproline, mg/100 g	49.34 ^d^	43.06	46.82	45.58	43.62	48.78	5.03	0.225	0.808	0.317
Cholesterol, mg/100 g	39.65 ^e^	40.22 ^e^	37.76	42.10	38.36	41.50	2.09	0.787	0.045	0.144
Backfat										
Cholesterol, mg/100 g	32.98 ^f^	29.90 ^f^	30.75	32.12	30.91	31.96	1.205	0.015	0.263	0.388

LW—Lithuanian White; LIW—Lithuanian Indigenous Wattle; CM—castrated males; F—females; Ad lib—ad libitum; R—restricted; SED—Standard error of difference; B—breed; G—gender; FR—feeding regimen. *p* values of GLM LSD tests for breed, gender, and feeding regimen and mean values within a columns between tissues with different superscriptsdiffer significantly ^a,b^ *p* ˂ 0.05; ^c,d^ *p* ˂ 0.01; ^e,f^ *p* ˂ 0.001.

**Table 6:** Effects of breed, gender, and feeding regimen during the finishing phase on the meat quality parameters.

Variables	Breed		Gender		Feeding Regimen		SED	*p*-Value		
	LW *n* = 24	LIW *n* = 18	CM *n* = 14	F *n* = 28	Ad lib *n* = 21	R *n* = 21		B	G	FR
Longissimus muscle										
pH1	6.23	6.32 ^e^	6.33	6.21	6.26	6.28	0.062	0.172	0.062	0.822
pH2	5.43 ^e^	5.46	5.46	5.43	5.43	5.46	0.032	0.450	0.416	0.439
Color: L *	54.48 ^e^	55.53	55.10 ^e^	54.91	55.22	54.79	0.749	0.172	0.798	0.565
A *	15.66 ^e^	14.91	15.33 ^e^	15.24	15.37	15.20	0.328	0.027	0.782	0.611
B *	7.13 ^a^	7.39	7.32	7.20	7.49	7.03	0.328	0.427	0.734	0.176
C	17.24 ^e^	16.66 ^e^	17.03	16.87	17.13	16.77	0.332	0.093	0.645	0.280
h	24.58 ^e^	26.30 ^e^	25.50	25.37	26.01	24.87	1.070	0.118	0.904	0.296
Drip loss,%	4.15	5.30 ^e^	4.40	5.04	4.55	4.89	0.703	0.113	0.369	0.628
Thawing loss,%	8.37	8.92	8.62	8.67	8.92	8.37	0.630	0.385	0.935	0.390
Cooking loss,%	30.89 ^e^	31.50 ^e^	31.03	31.37	30.77	31.63	0.841	0.474	0.692	0.317
Work of shear Nm	1.44	1.31 ^e^	1.34	1.40	1.37	1.38	0.070	0.085	0.396	0.815
Toughness, N	84.84	80.52 ^c^	80.62	84.74	80.65	84.71	4.869	0.381	0.404	0.410
Semimembranosus muscle										
pH1	6.25	6.60 ^f^	6.47	6.37	6.40	6.45	0.084	0.000	0.231	0.584
pH2	5.55 ^f^	5.55	5.57	5.53	5.53	5.56	0.043	0.869	0.304	0.523
Color: L *	46.93 ^f^	49.10 ^f^	48.29	47.74	48.61	47.42	1.215	0.084	0.655	0.335
A *	18.16 ^f^	17.01 ^f^	17.44	17.74	17.41	17.77	0.402	0.007	0.455	0.384
B *	6.73 ^b^	6.89	6.97	6.64	6.86	6.76	0.352	0.643	0.353	0.788
C	19.35 ^f^	18.39 ^f^	18.77	18.97	18.75	18.99	0.399	0.021	0.620	0.539
h	20.39 ^f^	22.10 ^f^	21.94	20.55	21.65	20.84	1.081	0.123	0.204	0.457
Drip loss,%	3.95	3.19	3.82	3.32	3.65	3.49	0.774	0.333	0.522	0.842
Thawing loss,%	9.03	7.32	8.16	8.19	8.36	7.99	0.935	0.078	0.976	0.689
Cooking loss,%	34.79 ^f^	35.54 ^f^	35.13	35.21	35.24	35.09	0.719	0.309	0.915	0.841
Work of shear Nm	1.53	1.63 ^f^	1.53	1.63	1.56	1.60	0.100	0.313	0.314	0.647
Toughness N	92.73	97.98 ^d^	92.53	98.18	93.53	97.18	8.102	0.522	0.491	0.655

LW—Lithuanian White; LIW—Lithuanian Indigenous Wattle; CM—castrated males; F—females; Ad lib-ad libitum; R—restricted; SED—Standard error of difference; B—breed; G—gender; FR—feeding regimen. *p* values of GLM LSD tests for breed, gender, feeding regimen and mean values within a columns between tissues with different superscripts differ significantly at ^a,b^ *p* ˂ 0.05; ^c,d^ *p* ˂ 0.01; ^e,f^ *p* ˂ 0.001.

**Table 7:** Total saturated, monounsaturated, polyunsaturated fatty acids (in %) and fatty acid ratios in intramuscular fat of muscles and backfat.

Variables	Breed		Gender		Feeding Regimen		SED	*p*-Value			
	LW *n* = 24	LIW *n* = 18	CM *n* = 14	F *n* = 28	Ad lib *n* = 21	R *n* = 21		B	G	FR	B × G
Longissimus muscle											
SFA	36.30	36.24	36.93	35.61	36.61	35.93	0.618	0.915	0.039	0.283	0.008
MUFA	51.92	52.34	52.62	51.63	51.57	52.69	1.188	0.728	0.410	0.352	0.537
PUFA	9.08	8.69	8.01	9.76	9.05	8.73	1.121	0.733	0.129	0.775	0.111
n-3 PUFA	1.04	0.93	0.89	1.08	1.01	0.97	0.128	0.390	0.143	0.770	0.129
n-6 PUFA	8.04	7.76	7.12	8.68	8.04	7.76	0.997	0.784	0.128	0.776	0.110
PUFA/SFA	0.25	0.25	0.22	0.28	0.25	0.25	0.036	0.808	0.121	0.897	0.076
n-6/n-3	7.72	8.33	8.03	8.03	8.04	8.01	0.227	0.010	1.000	0.893	0.965
Semimembranosus muscle											
SFA	32.64	35.28	35.46	32.46	34.47	33.45	0.649	0.000	0.000	0.126	0.022
MUFA	48.02	51.24	52.14	47.11	50.69	48.57	1.808	0.083	0.009	0.250	0.953
PUFA	14.66	10.38	9.62	15.43	11.38	13.67	1.623	0.013	0.001	0.168	0.547
n-3 PUFA	1.49	1.07	1.02	1.53	1.17	1.39	0.147	0.007	0.001	0.137	0.078
n-6 PUFA	13.17	9.32	8.60	13.89	10.21	12.28	1.480	0.014	0.001	0.172	0.100
PUFA/SFA	0.46	0.31	0.28	0.48	0.34	0.43	0.058	0.016	0.002	0.163	0460
n-6/n-3	8.73	8.45	8.21	8.97	8.55	8.63	0.260	0.301	0.007	0.770	0.622
Backfat											
SFA	42.62	43.34	43.95	42.01	43.43	42.53	0.738	0.336	0.013	0.233	0.123
MUFA	49.06	48.99	48.68	49.37	48.79	49.26	0.591	0.903	0.250	0.439	0.045
PUFA	7.97	7.37	7.04	8.29	7.47	7.87	0.467	0.208	0.011	0.398	0.844
n-3 PUFA	1.08	0.89	0.90	1.07	0.96	1.01	0.061	0.004	0.009	0.485	0.784
n-6 PUFA	6.89	6.48	6.14	7.22	6.50	6.86	0.410	0.324	0.013	0.390	0.637
PUFA/SFA	0.19	0.17	0.16	0.20	0.17	0.19	0.014	0.257	0.011	0.296	0.769
n-6/n-3	6.36	7.37	6.91	6.81	6.82	6.91	0.156	0.000	0.515	0.562	0.018

LW—Lithuanian White; LIW—Lithuanian Indigenous Wattle; CM—castrated males; F—females; Ad lib—ad libitum; R—restricted; SED—Standard error of difference; B—breed; G—gender; FR—feeding regimen; SFA = sum of all identified saturated fatty acids; MUFA = sum of all identified monounsaturated fatty acids; PUFA = sum of all identified polyunsaturated fatty acids; n-3 PUFA = sum of all identified n-3 polyunsaturated fatty acids; n-6 PUFA = sum of all identified n-6 polyunsaturated fatty acids. PUFA/SFA = ratio of ΣPUFA to ΣSFA, n-6/n-3 = ratio of Σn-6 PUFA to Σn-3 PUFA. *p*-values of GLM LSD tests for breed, gender, and feeding regimen were significantly different at *p* ˂ 0.05.

**Table 8:** Lipid quality indices in the intramuscular fat of muscles and backfat.

Variables	Breed		Gender		Feeding Regimen		SED	*p*-Value			
	LW *n* = 24	LIW *n* = 18	CM *n* = 14	F *n* = 28	Ad lib *n* = 21	R *n* = 21		B	G	FR	B × G
Longissimus muscle											
AI	0.46	0.47	0.48	0.46	0.48	0.46	0.011	0.313	0.076	0.188	0.007
TI	1.07	1.08	1.11	1.04	1.09	1.06	0.031	0.774	0.033	0.331	0.008
h/H	2.27	2.23	2.20	2.30	2.21	2.29	0.054	0.407	0.059	0.193	0.007
PI	19.58	18.94	17.48	21.04	19.67	18.84	2.574	0.806	0.175	0.748	0.106
Semimembranosus muscle											
AI	0.40	0.45	0.45	0.40	0.44	0.42	0.011	0.000	0.000	0.102	0.001
TI	0.91	1.04	1.04	0.90	0.99	0.95	0.030	0.000	0.000	0.116	0.000
h/H	2.64	2.34	2.35	2.62	2.43	2.54	0.066	0.000	0.000	0.108	0.003
PI	30.67	21.70	19.81	32.56	23.55	28.81	3.624	0.018	0.001	0.156	0.126
Backfat											
AI	0.54	0.55	0.56	0.53	0.56	0.53	0.013	0.465	0.011	0.074	0.043
TI	1.34	1.40	1.43	1.31	1.39	1.35	0.042	0.110	0.006	0.256	0.140
h/H	2.00	1.94	1.90	2.04	1.93	2.01	0.049	0.279	0.007	0.081	0.054
PI	11.20	10.89	10.13	11.96	10.81	11.28	0.615	0.615	0.006	0.447	0.783

LW—Lithuanian White; LIW—Lithuanian Indigenous Wattle; CM—castrated males; F—females; Ad lib—ad libitum; R—restricted; SED—Standard error of difference; B—breed; G—gender; FR—feeding regimen. PUFA/SFA = ratio of ΣPUFA to ΣSFA, n-6/n-3 = ratio of Σn-6 PUFA to Σn-3 PUFA, AI = atherogenic index, TI = thrombogenic index, h/H = hypocholesterolemic/hypercholesterolemic ratio, PI = peroxidizability index. *p* values of GLM LSD tests for breed, gender, and feeding regimen were significantly different at *p* ˂ 0.05.

## Data Availability

Data is contained within this article and supplementary material.

## Supplementary Materials

The following are available online at https://www.mdpi.com/article/10.3390/ani11041103/s1, Table S1. Fatty acid composition of intramuscular fat in longissimus muscle. Table S2. Fatty acid composition of intramuscular fat in semimembranosus muscle. Table S3. Fatty acid composition in subcutaneous tissue
