# Supplementary material for: Pork Quality of Two Lithuanian Breeds: Effects of Breed, Gender and Feeding Regimen
Source: Animals (Basel). 2021 Apr 12;11(4):1103. doi: 10.3390/ani11041103 (PMC8069488; doi:10.3390/ani11041103)
Supplement: Supplementary file 1 [file animals-11-01103-s001.pdf]

animals

MDPI

Table S1. Fatty acid composition of intramuscular fat in longissimus muscle.

Fatty acids	Breed		Gender		Feeding regimen		SED	p-Value		
	LW	LIW	CM	F	Ad lib	R		B	G	FR
C10:0	0.10	0.10	0.10	0.10	0.10	0.10	0.005	0.404	0.525	0.602
C12:0	0.07	0.07	0.07	0.07	0.07	0.07	0.003	0.397	0.786	0.371
C14:0	1.15	1.20	1.19	1.16	1.19	1.16	0.046	0.272	0.584	0.506
C15:0	0.04	0.05	0.04	0.05	0.05	0.04	0.006	0.582	0.427	0.770
C16:0	23.54	24.02	24.16	23.39	23.99	23.57	0.397	0.241	0.060	0.296
C17:0	0.20	0.18	0.19	0.19	0.19	0.19	0.013	0.159	0.887	0.748
C18:0	10.82	10.27	10.81	10.28	10.67	10.43	0.281	0.059	0.068	0.407
C20:0	0.16	0.15	0.16	0.14	0.15	0.15	0.013	0.524	0.199	0.803
C21:0	0.06	0.06	0.06	0.06	0.06	0.06	0.007	0.730	0.988	0.501
C22:0	0.16	0.14	0.14	0.16	0.14	0.15	0.024	0.525	0.556	0.714
C14:1n-7	0.02	0.02	0.02	0.01	0.03	0.01	0.005	0.393	0.049	0.022
C16:1n-7t	0.00	0.00	0.00	0.00	0.00	0.00	0.005	0.514	0.514	0.514
C16:1n-9	0.27	0.25	0.24	0.27	0.26	0.26	0.016	0.245	0.082	0.950
C16:1n-7	3.72	3.92	3.89	3.75	3.86	3.78	0.164	0.241	0.428	0.656
C17:1n-9	0.20	0.07	0.12	0.15	0.13	0.15	0.018	0.000	0.110	0.269
C18:1n-9t	0.20	0.19	0.19	0.19	0.19	0.20	0.007	0.089	0.991	0.075
C18:1n-9	42.08	42.32	42.62	41.78	41.71	42.69	1.032	0.819	0.419	0.350
C18:1n-7	4.70	4.80	4.75	4.75	4.69	4.81	0.135	0.508	0.987	0.357
C20:1n-9	0.73	0.78	0.79	0.72	0.72	0.79	0.041	0.274	0.100	0.094
C22:1n-9	0.00	0.00	0.00	0.00	0.00	0.00	0.002	0.441	0.441	0.441
C18:2n-6t9,12	0.05	0.05	0.05	0.05	0.05	0.05	0.007	0.953	0.795	0.350
C18:2n-6c9,t12	0.00	0.00	0.00	0.00	0.00	0.00	0.001	0.409	0.409	0.897
C18:2n-6t9,c12	0.00	0.00	0.01	0.01	0.00	0.00	0.001	0.572	0.572	0.572
C18:2n-6	5.90	5.63	5.18	6.35	5.85	5.68	0.667	0.689	0.089	0.804
C18:3n-6	0.07	0.07	0.06	0.07	0.07	0.06	0.013	0.995	0.323	0.476
C18:3n-3	0.45	0.38	0.40	0.44	0.42	0.41	0.033	0.046	0.211	0.572
C20:2n-6	0.14	0.14	0.13	0.15	0.13	0.14	0.009	0.546	0.026	0.428
C20:3n-6	0.22	0.22	0.19	0.24	0.22	0.21	0.037	0.938	0.181	0.703
C20:3n-3	0.10	0.09	0.08	0.11	0.10	0.09	0.011	0.698	0.041	0.785
C20:4n-6	1.43	1.41	1.29	1.56	1.46	1.39	0.267	0.949	0.324	0.780
C20:5n-3	0.14	0.11	0.11	0.14	0.13	0.12	0.026	0.335	0.285	0.781
C22:2n-6	0.02	0.00	0.01	0.01	0.01	0.01	0.005	0.001	0.706	0.969
C22:4n-6	0.22	0.25	0.22	0.25	0.25	0.22	0.033	0.313	0.343	0.300
C22:5n-3	0.31	0.28	0.26	0.33	0.30	0.29	0.051	0.649	0.162	0.830
C22:6n-3	0.06	0.07	0.05	0.07	0.07	0.06	0.019	0.604	0.205	0.762
Total trans	0.25	0.24	0.24	0.25	0.24	0.25	0.011	0.317	0.552	0.121
SFA	36.30	36.24	36.93	35.61	36.61	35.93	0.618	0.915	0.039	0.283
MUFA	51.92	52.34	52.62	51.63	51.57	52.69	1.188	0.728	0.410	0.352
PUFA	9.08	8.69	8.01	9.76	9.05	8.73	1.121	0.733	0.129	0.775
Unindentified FA	2.70	2.73	2.43	3.00	2.78	2.65	0.445	0.937	0.207	0.782

LW-Lithuanian White; LIW-Lithuanian Indigenous Wattle; CM-castrated males; F-females; Ad lib-ad libitum; R-restricted; SED-Standard error of difference; B-breed; G-gender; FR-feeding regimen. SFA = sum of all identified saturated fatty acids; MUFA.= sum of all identified monounsaturated fatty acids. PUFA = sum of all identified polyunsaturated fatty acids.UFA.= sum of all unidentified fatty acids. *p*values of GLM LSD tests for breed, gender and feeding regimen are significantly different at $p<0.05$

Table S2. Fatty acid composition of intramuscular fat in semimembranosus muscle.

**Fatty acids**	**Breed**		**Gender**		**Feeding regimen**		**SED**	**p-Value**		
	**LW**	**LIW**	**CM**	**F**	**Ad lib**	**R**		**B**	**G**	**FR**
C10:0	0.09	0.09	0.10	0.08	0.09	0.09	0.005	0.274	0.028	0.135
C12:0	0.06	0.07	0.00	0.00	0.07	0.06	0.003	0.207	0.006	0.051
C14:0	0.97	1.13	1.13	0.97	1.09	1.01	0.049	0.002	0.003	0.087
C15:0	0.06	0.04	0.05	0.06	0.05	0.06	0.006	0.003	0.253	0.297
C16:0	21.04	23.34	23.19	21.18	22.55	21.82	0.430	0.000	0.000	0.101
C17:0	0.23	0.20	0.21	0.22	0.21	0.22	0.016	0.030	0.740	0.418
C18:0	9.73	10.07	10.34	9.46	10.02	9.78	0.257	0.184	0.002	0.346
C20:0	0.12	0.13	0.14	0.11	0.13	0.12	0.010	0.513	0.004	0.463
C21:0	0.06	0.06	0.06	0.06	0.06	0.06	0.008	0.514	0.792	0.573
C22:0	0.29	0.15	0.18	0.26	0.20	0.24	0.040	0.002	0.036	0.269
C14:1n-7	0.00	0.01	0.01	0.00	0.01	0.00	0.002	0.000	0.000	0.002
C16:1n-7t	0.00	0.003	0.00	0.003	0.00	0.00	0.005	0.514	0.514	0.514
C16:1n-9	0.33	0.27	0.27	0.33	0.30	0.30	0.015	0.000	0.000	0.636
C16:1n-7	3.50	3.77	3.84	3.43	3.77	3.49	0.170	0.114	0.022	0.109
C17:1n-9	0.13	0.11	0.10	0.14	0.10	0.14	0.022	0.501	0.050	0.093
C18:1n-9t	0.23	0.20	0.22	0.20	0.21	0.22	0.013	0.015	0.153	0.503
C18:1n-9	38.26	41.29	41.99	37.56	40.66	38.89	1.581	0.064	0.008	0.272
C18:1n-7	4.91	4.84	4.92	4.82	4.92	4.83	0.153	0.613	0.519	0.557
C20:1n-9	0.66	0.76	0.80	0.63	0.72	0.70	0.044	0.029	0.002	0.633
C18:2n-6t9,12	0.04	0.05	0.04	0.05	0.04	0.05	0.010	0.601	0.572	0.412
C18:2n-6c9,t12t	0.00	0.00	0.00	0.00	0.00	0.00	0.002	0.346	0.346	0.346
C18:2n-6t9,c12	0.00	0.00	0.00	0.00	0.00	0.00	0.002	0.441	0.441	0.441
C18:2n-6	9.25	6.65	6.23	9.66	7.29	8.60	0.948	0.010	0.001	0.174
C18:3n-6	0.09	0.07	0.06	0.10	0.07	0.09	0.016	0.172	0.005	0.355
C18:3n-3	0.55	0.42	0.44	0.53	0.47	0.50	0.034	0.001	0.010	0.536
C20:2n-6	0.19	0.16	0.15	0.19	0.16	0.18	0.011	0.005	0.001	0.065
C20:3n-6	0.37	0.25	0.23	0.39	0.28	0.34	0.051	0.020	0.002	0.279
C20:3n-3	0.18	0.12	0.12	0.18	0.14	0.16	0.018	0.003	0.002	0.308
C20:4n-6	2.89	1.84	1.60	3.13	2.06	2.67	0.444	0.024	0.002	0.185
C20:5n-3	0.23	0.14	0.13	0.24	0.16	0.21	0.034	0.008	0.005	0.130
C22:2n-6	0.01	0.00	0.01	0.00	0.00	0.01	0.006	0.050	0.627	0.415
C22:4n-6	0.33	0.31	0.28	0.35	0.29	0.35	0.042	0.624	0.075	0.293
C22:5n-3	0.46	0.34	0.30	0.50	0.35	0.45	0.060	0.046	0.002	0.114
C22:6n-3	0.07	0.05	0.04	0.09	0.05	0.08	0.015	0.172	0.002	0.030
TFA	0.27	0.25	0.27	0.26	0.25	0.27	0.017	0.169	0.567	0.439
SFA	32.64	35.28	35.46	32.46	34.47	33.45	0.649	0.000	0.000	0.126
MUFA	48.02	51.24	52.14	47.11	50.69	48.57	1.808	0.083	0.009	0.250
PUFA	14.66	10.38	9.62	15.43	11.38	13.67	1.623	0.013	0.001	0.168
UFA	4.68	3.10	2.78	5.00	3.47	4.31	0.618	0. 015	0.001	0.181

LW-Lithuanian White; LIW-Lithuanian Indigenous Wattle; CM-castrated males; F-females; Ad lib-ad libitum; R-restricted; SED-Standard error of difference; B-breed; G-gender; FR-feeding regimen;TFA = sum of all identified *trans* fatty acids; SFA = sum of all identified saturated fatty acids; MUFA.= sum of all identified monounsaturated fatty acids; PUFA = sum of all identified polyunsaturated fatty acids; UFA.= sum of all unidentified fatty acids. *p*values of GLM LSD tests for breed, gender and feeding regimen are significantly different at $p<0.05$

Table S3. Fatty acid composition in subcutaneous tissue

Fatty acids	Breed		Gender		Feeding regimen		SED	p-Value		
	LW	LIW	CM	F	Ad lib	R		B	G	FR
C10:0	0.07	0.05	0.06	0.06	0.06	0.06	0.003	0.000	0.633	0.205
C12:0	0.07	0.06	0.07	0.07	0.07	0.07	0.002	0.000	0.561	0.095
C14:0	1.31	1.24	1.27	1.28	1.31	1.24	0.037	0.053	0.788	0.096
C15:0	0.04	0.03	0.03	0.04	0.04	0.04	0.003	0.000	0.153	0.970
C16:0	25.42	25.84	26.09	25.17	25.96	25.29	0.337	0.214	0.010	0.054
C17:0	0.31	0.25	0.28	0.28	0.27	0.29	0.022	0.007	0.880	0.573
C18:0	15.01	15.52	15.76	14.77	15.36	15.18	0.558	0.361	0.085	0.747
C20:0	0.25	0.26	0.28	0.24	0.26	0.26	0.012	0.682	0.002	0.899
C21:0	0.09	0.05	0.07	0.07	0.06	0.07	0.007	0.000	0.541	0.070
C22:0	0.05	0.04	0.04	0.04	0.04	0.05	0.004	0.003	0.860	0.523
C14:1n-7	0.00	0.00	0.00	0.00	0.00	0.00	0.002	0.048	0.276	0.048
C16:1n-7tr	0.02	0.02	0.02	0.02	0.02	0.02	0.003	0.251	0.232	0.316
C16:1n-9	0.30	0.28	0.26	0.32	0.27	0.31	0.025	0.296	0.020	0.164
C16:1n-7	1.96	1.95	1.97	1.95	2.00	1.92	0.115	0.932	0.096	0.476
C18:1n-9tr	0.32	0.29	0.31	0.30	0.29	0.32	0.020	0.105	0.792	0.096
C18:1n-9	42.32	42.19	41.90	42.61	42.09	42.42	0.509	0.791	0.176	0.516
C18:1n-7	2.94	2.89	2.88	2.95	2.89	2.94	0.120	0.678	0.577	0.646
C20:1n-9	1.18	1.36	1.33	1.21	1.23	1.31	0.058	0.002	0.040	0.144
C22:1n-9	0.01	0.01	0.01	0.01	0.01	0.01	0.003	0.879	0.175	0.512
C18:2n-6t9,12	0.06	0.05	0.05	0.05	0.05	0.06	0.006	0.039	0.880	0.421
C18:2n-6c9,t12	0.03	0.02	0.03	0.02	0.02	0.03	0.003	0.006	0.498	0.083
C18:2n-6t9,c12	0.04	0.02	0.03	0.03	0.03	0.06	0.005	0.028	0.937	0.142
C18:2n-6	6.18	5.67	5.43	6.42	5.77	6.08	0.382	0.195	0.014	0.434
C18:3n-6	0.02	0.02	0.02	0.03	0.02	0.02	0.006	0.826	0.110	0.496
C18:3n-3	0.84	0.63	0.68	0.79	0.73	0.75	0.042	0.000	0.018	0.636
C20:2n-6	0.35	0.37	0.35	0.37	0.34	0.38	0.017	0.203	0.137	0.026
C20:3n-6	0.05	0.05	0.05	0.06	0.05	0.05	0.004	0.772	0.045	0.365
C20:3n-3	0.15	0.15	0.14	0.16	0.14	0.16	0.007	0.627	0.012	0.044
C20:4n-6	0.12	0.15	0.12	0.14	0.13	0.13	0.011	0.014	0.136	0.786
C20:5n-3	0.00	0.00	0.00	0.00	0.00	0.00	0.004	0.625	0.512	0.512
C22:4n-6	0.05	0.13	0.08	0.11	0.09	0.09	0.024	0.002	0.214	0.810
C22:5n-3	0.08	0.08	0.07	0.09	0.08	0.08	0.009	0.725	0.073	0.970
C22:6n-3	0.01	0.03	0.01	0.03	0.02	0.02	0.017	0.171	0.154	0.806
Total trans	0.46	0.40	0.43	0.43	0.40	0.46	0.030	0.032	0.896	0.085
SFA	42.62	43.34	43.95	42.01	43.43	42.53	0.738	0.336	0.013	0.233
MUFA	49.06	48.99	48.68	49.37	48.79	49.26	0.591	0.903	0.250	0.439
PUFA	7.97	7.37	7.04	8.29	7.47	7.87	0.467	0.208	0.011	0.398
UFA	0.35	0.31	0.33	0.33	0.31	0.35	0.028	0.110	0.852	0.234

LW-Lithuanian White; LIW-Lithuanian Indigenous Wattle; CM-castrated males; F-females; Ad lib-ad libitum; R-restricted; SED-Standard error of difference; B-breed; G-gender; FR-feeding regimen; TFA = sum of all identified *trans* fatty acids; SFA = sum of all identified saturated fatty acids; MUFA= sum of all identified monounsaturated fatty acids; PUFA = sum of all identified polyunsaturated fatty acids; UFA = sum of all unidentified fatty acids. *p* values of GLM LSD tests for breed, gender and feeding regimen are significantly different at $p<0.05$
